# miR-454 functions as an oncogene by inhibiting CHD5 in hepatocellular carcinoma

**DOI:** 10.18632/oncotarget.4407

**Published:** 2015-07-30

**Authors:** Lei Yu, Xuejun Gong, Lei Sun, Hong Yao, Baoling Lu, Liying Zhu

**Affiliations:** ^1^ Department of Infectious Disease, The Fourth Hospital of Harbin Medical University, Harbin 150001, Heilongjiang, China; ^2^ Department of Biliary and Pancreatic Surgery, Xiangya Hospital of Central South University, Changsha, 410008, Hunan, China; ^3^ Department of Ophthalmology, The Fourth Hospital of Harbin Medical University, Harbin 150001, Heilongjiang, China

**Keywords:** hepatocellular carcinoma, microRNA, miR-454, CHD5

## Abstract

Previous studies showed that miR-454 acted as an oncogene or tumor suppressor in cancer. However, its function in HCC remains unknown. In this study, we found that miR-454 expression was upregulated in HCC cell lines and tissues. Knockdown of miR-454 inhibited HCC cell proliferation and invasion and epithelial mesenchymal transition (EMT), whereas overexpression of miR-454 promoted HCC cell proliferation and invasion and EMT. Furthermore, we identified the CHD5 as a direct target of miR-454. CHD5 was downregulated in HCC tissues and cell lines and the expression level of CHD5 was inversely correlated with the expression of miR-454 in HCC tissues. In addition, knockdown of miR-454 inhibited the growth of HepG2-engrafted tumors *in vivo*. Taken together, these results indicated that miR-454 functioned as an oncogene in HCC.

## INTRODUCTION

Hepatocellular carcinoma (HCC), the primary cancer of liver, ranks as the fifth most frequent malignancy worldwide, accounting for ∼700 000 deaths per year [1–3]. Although various genetic and epigenetic changes leading to HCC have been revealed, the prognosis of HCC has not been improved [4–7]. In the past decades, many studies have identified factors associated with HCC survival; however, the 5-year survival rate remains quite low among patients with HCC [8–10]. There is an urgent need to find specific and effective diagnostic biomarkers for early stage HCC.

MicroRNAs (miRNAs) are endogenous non-coding RNAs (about 22 nt) that have drawn attention in recent years as one of the key modulators [11–16]. MiRNAs interact with various targets and regulate many cellular processes by inhibiting the expression of downstream genes through base pairing with the 3′-ntranslated region (3′-UTR) of the corresponding mRNAs [17–20]. Increasing evidences have indicated that miRNAs play important roles in cell proliferation, apoptosis, migration, invasion and metabolism [21–24]. miRNAs may function as tumor suppressor genes or oncogenes, depending on the target genes [25–29]. Recent reports have indicated that deregulation of miRNAs is associated with the formation and progression of many cancers such as gastric cancer, bladder cancer, breast cancer and HCC [30–34]. Therefore, miRNAs are potentially useful biomarkers for clinical diagnosis.

In this study, we demonstrated that miR-454 expression was upregulated in human cell lines and HCC tissues compared with normal liver cell line and adjacent non-tumor tissue. Knockdown of miR-454 inhibited HCC cell proliferation and invasion and EMT. Furthermore, we identified the CHD5 (chromodomain–helicase–DNA–binding-5) as a direct target of miR-454. CHD5 was downregulated in HCC tissues and cell lines and the expression of CHD5 was inversely correlated with the expression of miR-454 in HCC tissues. In addition, knockdown of miR-454 inhibited the growth of HepG2-engrafted tumors *in vivo*.

## RESULTS

### miR-454 was elevated in HCC tissues and cell lines

**Figure 1:**
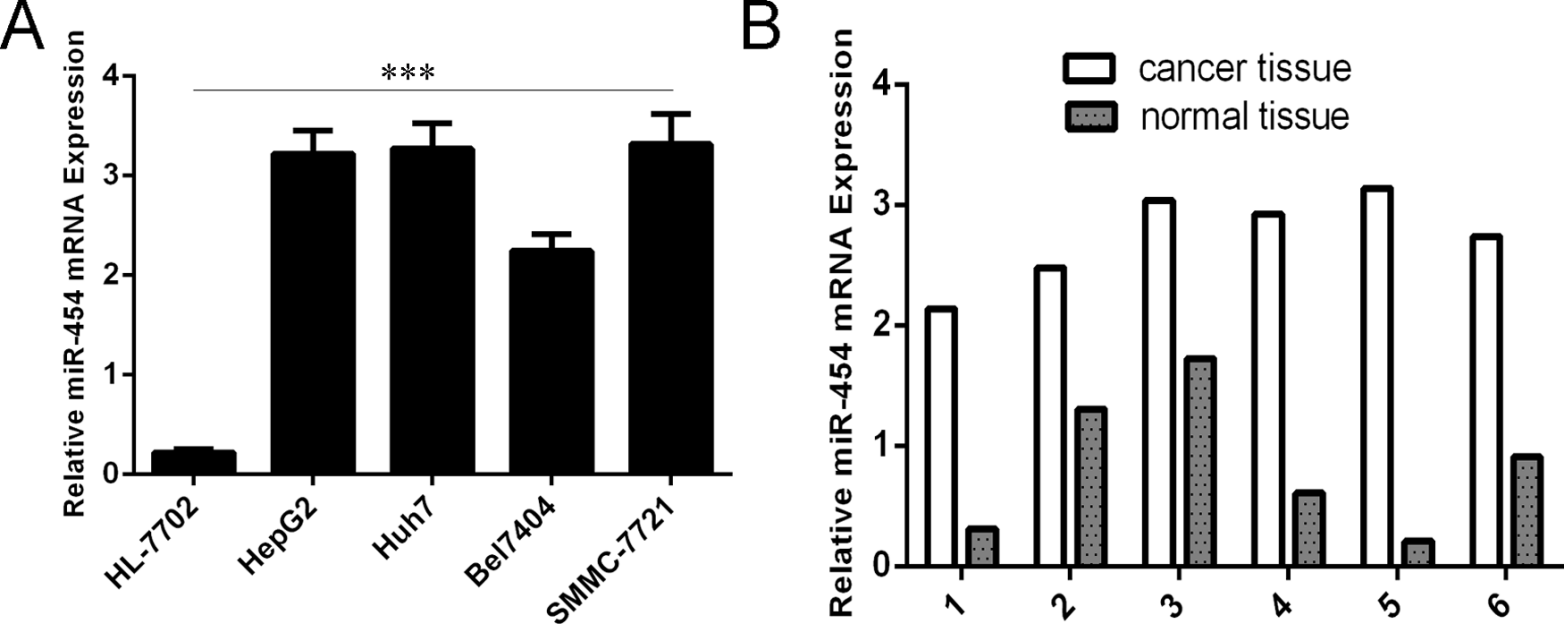
miR-454 was elevated in cell lines **A.** The expression of miR-454 in four HCC cell lines (SMMC-7721, Bel-7404, Huh7 and HepG2) and one normal liver cell (HL-7702 cells) was measured by using qRT-PCR. **B.** The expression of miR-454 in six HCC tissues and adjacent non-tumor tissues was measured by using qRT-PCR. The expression of miR-454 was normalized to U6 snRNA. ***p < 0.001.

**Figure 2:**
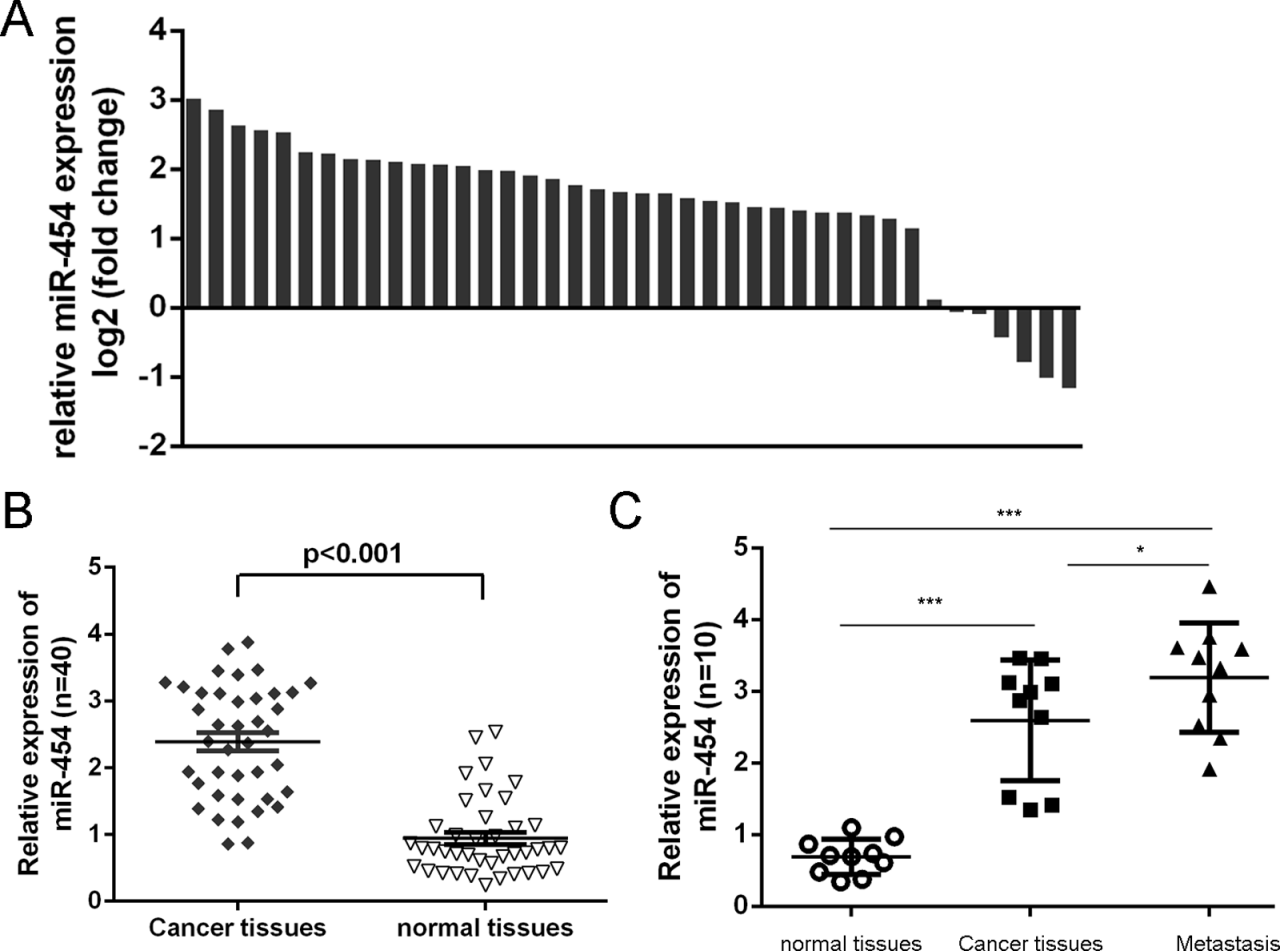
miR-454 was elevated in HCC tissues **A.** qRT-PCR analysis of miR-454 expression in 40 pairs HCC tissues and their corresponding nontumor tissues. The expression of miR-454 was normalized to U6 snRNA. **B.** The expression of miR-454 in HCC tissues was significant lower than in adjacent tissues. **C.** The expression of miR-454 in 10 pairs lymph node metastases, HCC tissues and their corresponding nontumor tissues. *p < 0.05, **p < 0.01, and ***p < 0.001.

As shown in Fig. 1A, the expression of miR-454 was upregulated in four HCC cell lines (SMMC-7721, Bel-7404, Huh7 and HepG2) compared with in one normal liver cell (HL-7702 cells). We also found that miR-454 expression was elevated in six HCC tissues compared with in adjacent non-tumor tissues (Fig. 1B). Moreover, the increase of miR-454 was found in 34 of 40 HCC tissues compared with the corresponding non-tumor tissues and the expression level of miR-454 in HCC tissues was significant higher than in adjacent tissues (Fig. 2A and 2B, *p* < 0.001). Furthermore, lymph node metastases tissues expressed higher levels of miR-454 compared with the primary HCC tissues and the corresponding normal tissues (Fig. 2C).

### Knockdown of miR-454 inhibited HCC cell proliferation and invasion and epithelial mesenchymal transition (EMT)

**Figure 3:**
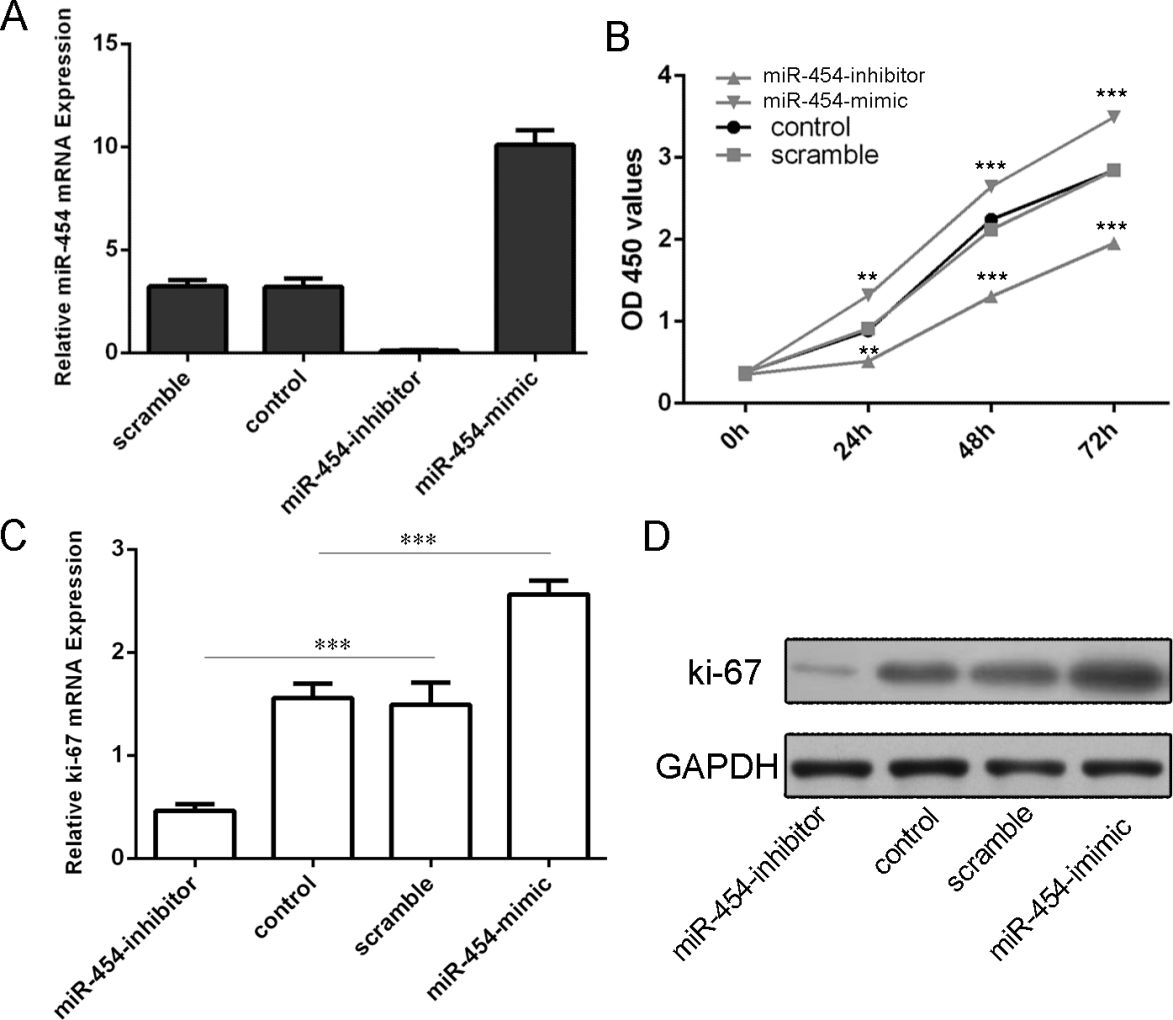
Knockdown of miR-454 inhibited HCC cell proliferation **A.** miR-454 mimics can enhance the expression of miR-454 and miR-454 inhibitor can repress the expression of miR-454 in the HepG2 cells. **B.** CCK-8 proliferation assay showed that overexpression of miR-454 significantly promoted the growth rate of cells compared with control cells in HepG2 cells. Conversely, miR-211 inhibitor significantly inhibited the proliferation of the HepG2 cells. **C.** The mRNA of Ki-67 was measured by using qRT-PCR. **D.** The protein of Ki-67 was measured by using western blot. *p < 0.05, **p < 0.01, and ***p < 0.001.

**Figure 4:**
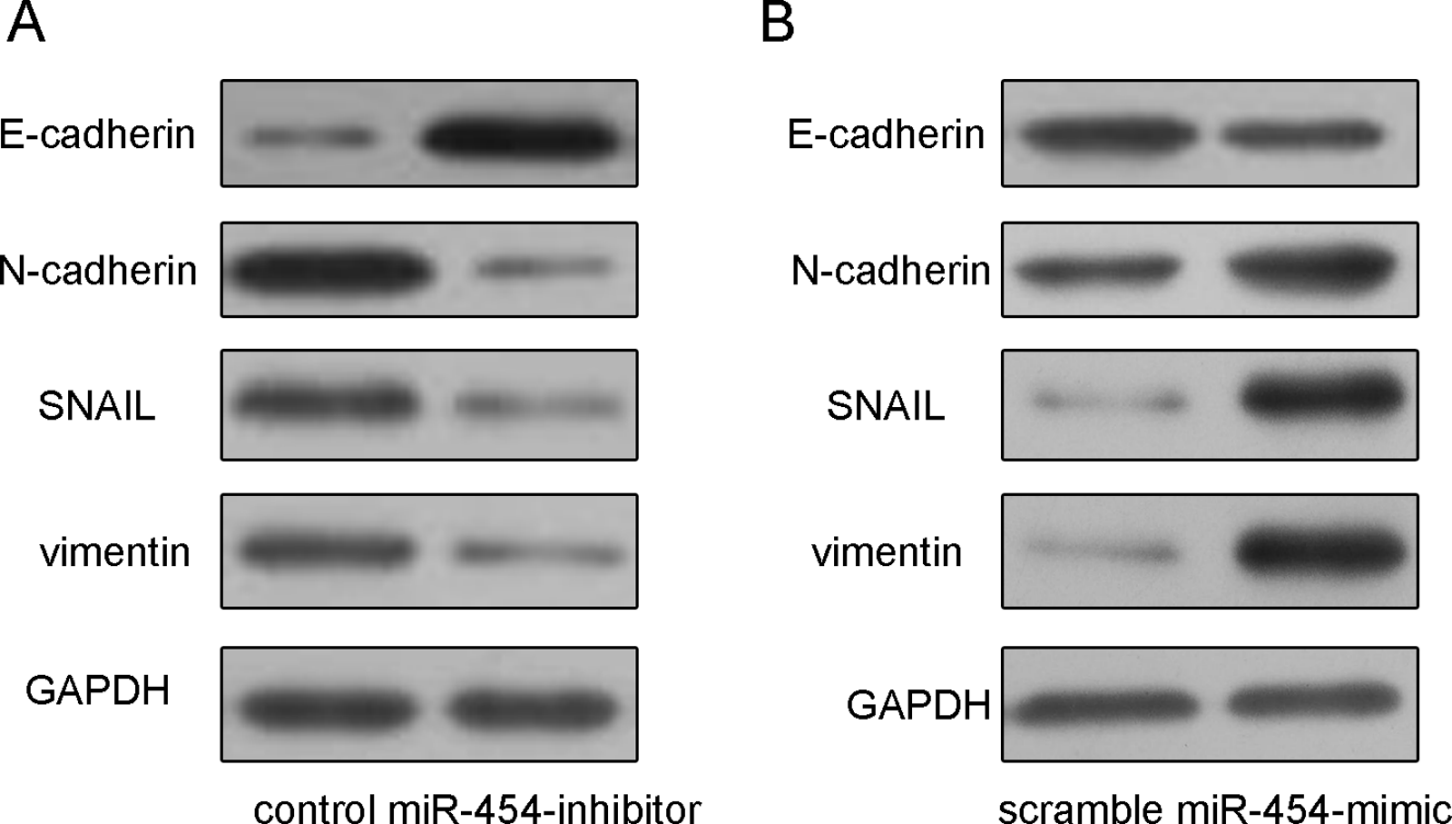
Knockdown of miR-454 inhibited HCC cell epithelial mesenchymal transition **A.** Inhibition of miR-454 increased the protein expression of E-cadherin and inhibited the N-cadherin, Snail and vimentin expression in HepG2 cells. **B.** Overexpression of miR-454 inhibited the protein expression of E-cadherin and increased the N-cadherin, Snail and vimentin expression.

**Figure 5:**
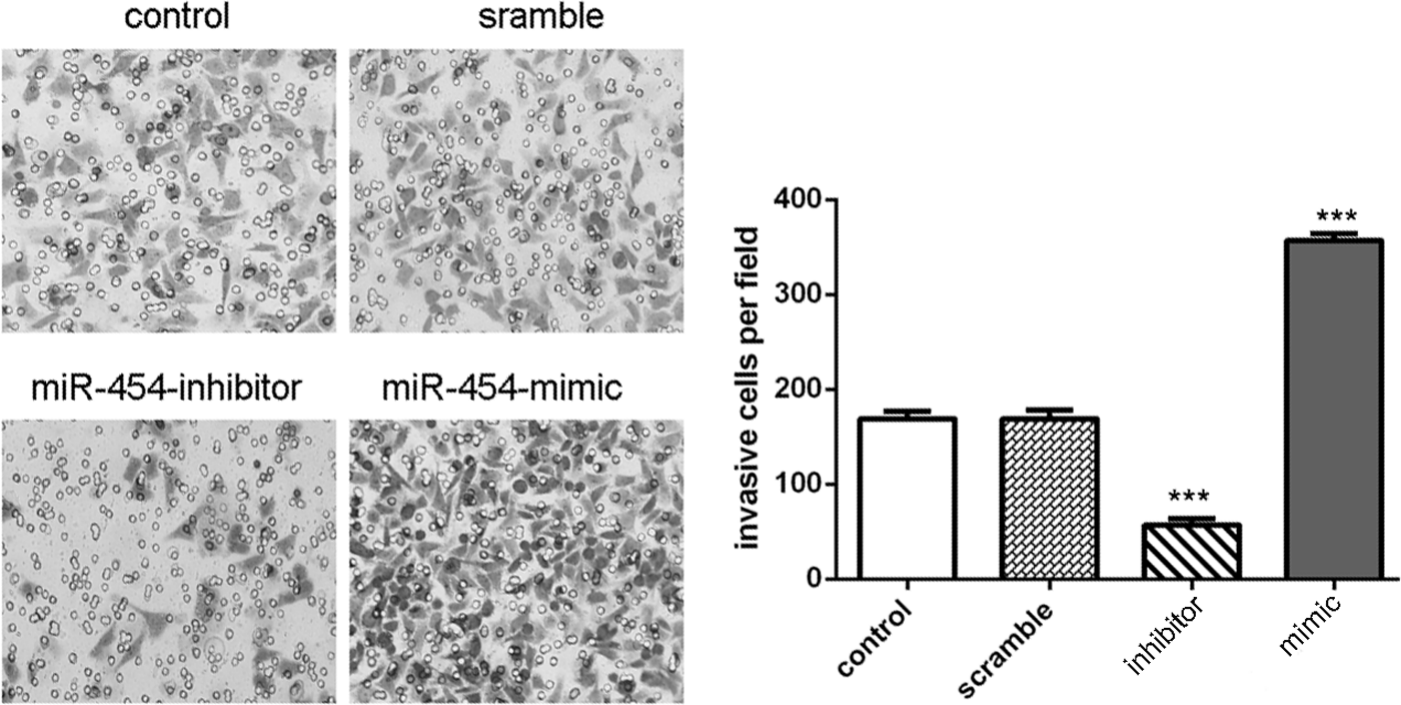
Knockdown of miR-454 inhibited invasion Knockdown of miR-454 inhibited the HepG2 cells invasion, whereas overexpression of miR-454 increased the HepG2 cells invasion. *p < 0.05, **p < 0.01, and ***p < 0.001.

The transfection efficiency was confirmed by qRT-PCR in HepG2 cells (Fig. 3A). Knockdown of miR-454 inhibited HepG2 cells proliferation, whereas overexpression of miR-454 promoted the HepG2 cells proliferation (Fig. 3B). Moreover, knockdown of miR-454 repressed the Ki-67 mRNA and protein expression, whereas overexpression of miR-454 increased the Ki-67 mRNA and protein expression (Fig. 3C and 3D). Inhibition of miR-454 increased the expression of E-cadherin and inhibited the N-cadherin, Snail and vimentin expression, whereas overexpression of miR-454 inhibited the expression of E-cadherin and increased the N-cadherin, Snail and vimentin expression (Fig. 4A and 4B). These results demonstrated that knockdown of miR-454 inhibited epithelial mesenchymal transition (EMT). cells invasion, whereas overexpression of miR-454 increased cells invasion (Fig. 5).

### CHD5 was a direct target of miR-454

**Figure 6:**
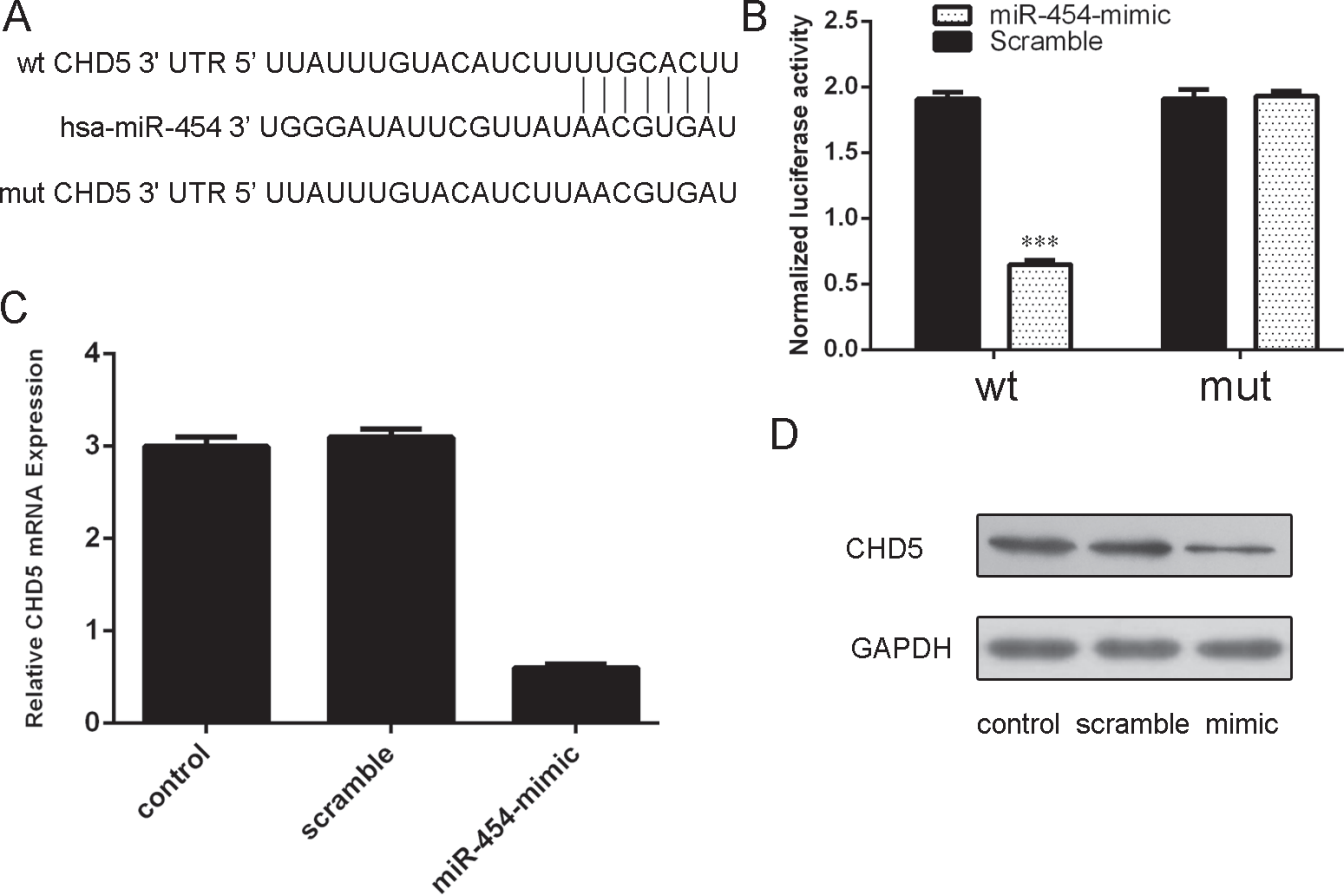
CHD5 was a direct target of miR-454 **A.** Predicted miR-454 target sequence in the 3′UTR of CHD5 and mutant containing 7 altered nucleotides in the 3′UTR of CHD5. **B.** The analysis of the relative luciferase activities of CHD5-WT, CHD5-MUT in the HepG2 cells. **C.** qRT-PCR analysis of CHD5 mRNA expression in the HepG2 cells after treatment with miRNA mimics or scramble or no transfection. The expression of CHD5 was normalized to GAPDH. **D.** Western blot analysis of CHD5 expression in the HepG2 cells transfected with miR-564 mimics or scramble or no transfection. GAPDH was also detected as a loading control.

CHD5 was proved to be a putative target gene of miR-454 by using database TargetScan (Fig. 6A). The direct effect of miR-454 on the translation of CHD5 mRNA into protein was measured by luciferase reporter assay in HepG2 cells (Fig. 6B). Ectopic expression of miR-454 inhibited the CHD5 mRNA and protein expression (Fig. 6C and 6D).

### CHD5 was downregulated in HCC tissue and cell lines and was inversely expressed with miR-454

**Figure 7:**
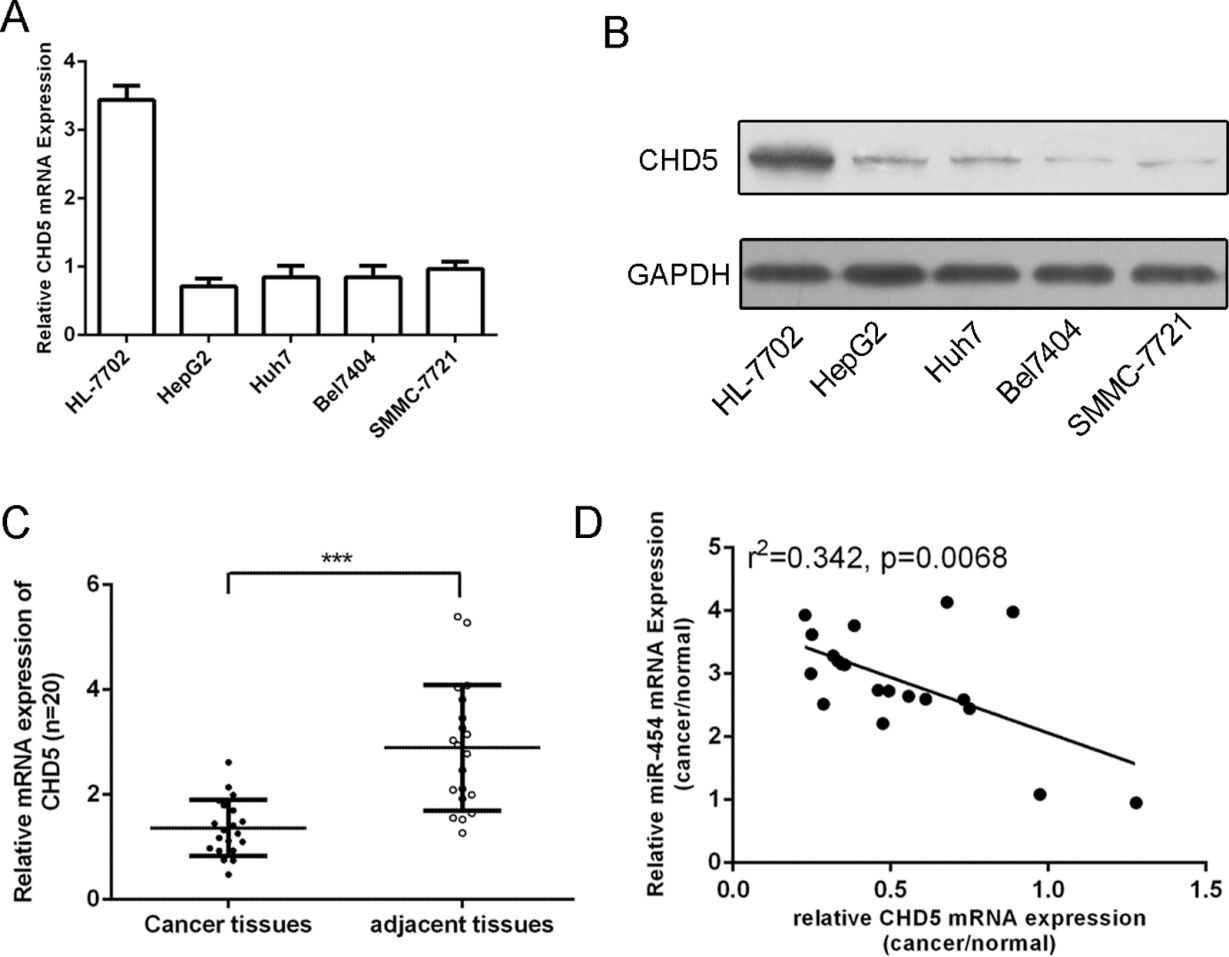
CHD5 was downregulated in HCC tissue and cell lines and was inversely expressed with miR-454 **A.** Expression levels of CHD5 in four cell lines (MHCC-97H, QGY-7703, SMMC7721 and HepG2) and one normal liver cell (HL-7702 cells) were detected using qRT-PCR analysis. **B.** The protein expression levels of CHD5 in four cell lines (MHCC-97H, QGY-7703, SMMC7721 and HepG2) and one normal liver cell (HL-7702 cells) were detected using western blot. **C.** The expression of CHD5 in HCC tissues was significant lower than in adjacent tissues. **D.** Comparison of miR-454 levels and levels corresponding to CHD5 in HCC exhibited significantly inverse correlation between CHD5 and miR-454 (r^2^ = 0.342, *P* = 0.0068). ***p < 0.001.

As shown in Fig. 7A and 7B, the mRNA and protein expression of CHD5 was downregulated in four HCC cell lines (SMMC-7721, Bel-7404, Huh7 and HepG2) compared with in one normal liver cell (HL-7702 cells). We also found that CHD5 expression was downregulated in HCC tissues compared with that in adjacent non-tumor tissues (Fig. 7C). In addition, expression levels of miR-454 inversely correlated with the expression levels of CHD5 in HCC (r^2^ = 0.342, *P* = 0.0068) (Fig. 7D).

### Knockdown of miR-454 inhibited the growth of HepG2-engrafted tumors

**Figure 8:**
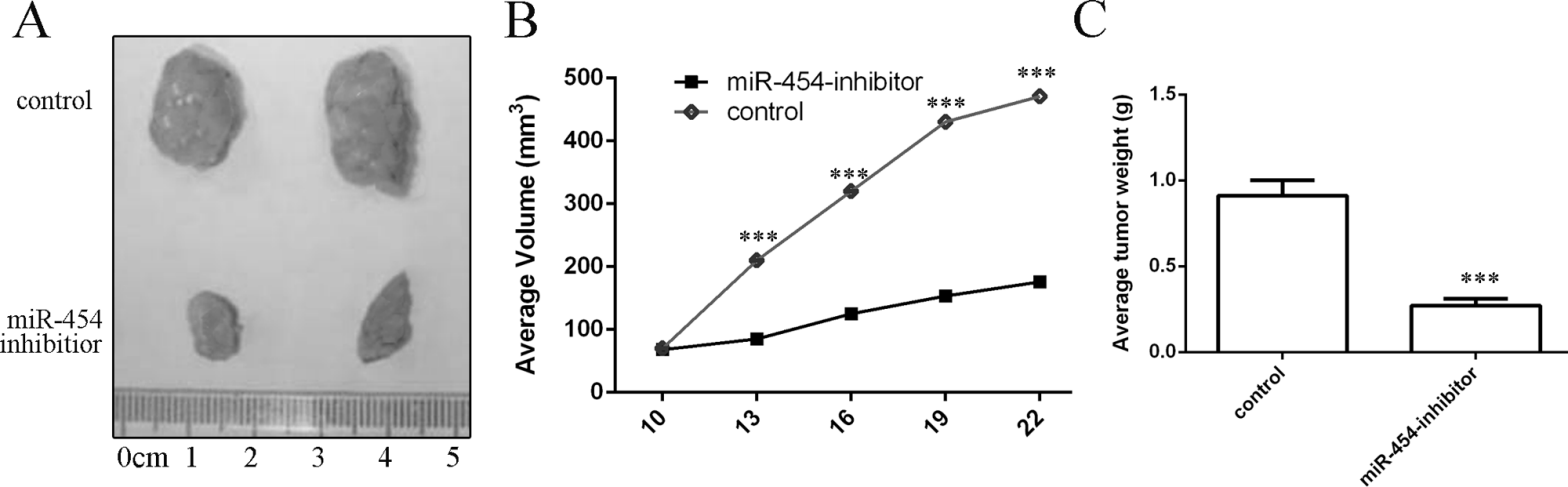
Knockdown of miR-454 inhibited the growth of HepG2-engrafted tumors **A.** Representative tumors were photographed at 15 days after the first treatment with miR-454 inhibitor or control. **B.** Graph representing tumor volumes at the indicated days during the experiment for the two groups: control, miR-454 inhibitor. **C.** Tumor weight averages between control and miR-454 inhibitor-treated mice groups at the end of the experiment (day 25), Five mice in each group.

miR-454 inhibitor inhibited the growth of HepG2-engrafted tumors compared to control oligonucleotides-treated tumors (Fig. 8A). The volumes and weights of tumors treated by miR-454 inhibitor were also lower than control oligonucleotides-treated tumors (Fig. 8B and 7C).

## DISCUSSION

In the recent years, deregulation of miRNAs has been a common event that can regulate cell proliferation and invasion in many cancers [35–37]. In our study, we showed that miR-454 expression was upregulated in human cell lines and HCC tissues compared with normal liver cell line and adjacent non-tumor tissue. Moreover, lymph node metastases tissues expressed higher levels of miR-454 compared with the primary HCC tissues and the corresponding normal tissues. Knockdown of miR-454 inhibited HCC cell proliferation and invasion and EMT, whereas overexpression of miR-454 promoted HCC cell proliferation and invasion and EMT. Furthermore, we identified the CHD5 as a direct target of miR-454. CHD5 was downregulated in HCC tissues and cell lines and the expression level of CHD5 was inversely correlated with the expression level of miR-454 in HCC tissues. In addition, knockdown of miR-454 inhibited the growth of HepG2-engrafted tumors *in vivo*. Taken together, these results indicated that miR-454 functioned as an oncogene, indicating their potential use in the therapy of HCC.

Recently, the role of miR-454 was reported in several tumors. For example, the miR-454 expression was increased in colorectal cancer (CRC) tissues and CRC cells[38]. Overexpression of miR-454 promoted the proliferation and anchorage-independent growth of CRC cells and its oncogenic effect was mediated chiefly through direct suppression of CYLD expression. Another study reported that miR-454 expression was depressed in activated rat hepatic stellate cells and miR-454 expression was decreased in LX-2 cells treated with TGF-β1 [38]. Perfetti et al. demonstrated that miR-454 expression was upregulated in the blood of myotonic dystrophy type 1 patients [39]. Niu et al. showed that miR-454 expression was downregulated in osteosarcoma tissues, acting as a tumor suppressor gene in osteosarcoma. However, the relationship between HCC and miR-454 expression is still unknown. In our study, we firstly measured the expression of miR-454 in HCC cell lines and tissues. We found that miR-454 expression was upregulated in human cell lines and HCC tissues. Moreover, knockdown of miR-454 inhibited HCC cell proliferation and invasion and EMT, whereas overexpression of miR-454 promoted HCC cell proliferation and invasion and EMT. Knockdown of miR-454 inhibited the growth of HepG2-engrafted tumors *in vivo*. These results indicated that miR-454 functioned as an oncogene in HCC.

CHD5 was a member of a 9 member family of CHD chromatin remodeling proteins, which was first identified in neuroblastomas on 1p36 in a region of most deletion [40, 41]. Previous studies demonstrated that CHD5 acted as tumor suppressor gene in a lot of cancers such as neuroblastoma, laryngeal squamous cell carcinoma, colon cancer, lung cancer and gastric cancer [42–47]. Zhao et al. also showed that CHD5 played a tumor suppressor gene role in the HCC [48]. They demonstrated that the expression CHD5 was downregulated in HCC cell lines and tissues, and the −841 to −470 region of CHD5 promoter was hypermethylated in these samples. Moreover, ectopic expression of CHD5 repressed cell proliferation, tumourigenicity and colony formation and lead cellular senescence. In our study, several experiments showed that miR-454 could directly bind to CHD5 mRNA and inhibited CHD5 expression. We performed the luciferase activity assay by transfecting miR-454 mimics into the HepG2 cells and found that miR-454 could bind to the 3′UTR of CHD5 and repress the luciferase activity. Moreover, overexpression of miR-454 inhibited the mRNA and protein of CHD5. In line with previous studies, we demonstrated the expression of CHD5 was downregulated in HCC cell lines and tissues and the expression of CHD5 was inversely correlated with the expression of miR-454 in HCC tissues. These results demonstrate that CHD5 is a direct target gene of miR-454 in development of HCC.

In conclusion, the present study provided miR-454 acted an oncogenic miRNA in the tumorigenesis and progression of HCC. This study was the first evidence of the overexpression and clinical significance of miR-454 in HCC, suggesting that miR-454 might serve as a valuable prognostic marker for HCC patients.

## MATERIALS AND METHODS

### Cell lines, cell culture, and human tissue samples

Human liver cancer cell line SMMC-7721, Bel-7404, Huh7, HepG2 and normal liver cell HL-7702 cells were maintained in 1640 medium (1640; PAA Laboratories GmbH) supplemented with 10% FBS (PAA Laboratories GmbH). Forty paired hepatocellular carcinoma and adjacent nontumor liver tissues (located > 3 cm from the tumor) were collected from patients undergoing resection of hepatocellular carcinoma at our hospital. The relevant characteristics of the studied subjects were shown in Table S1. No systemic or local treatment had been received before operation. Both nontumor and tumor tissues were histologically confirmed. Informed consent was obtained from each patient and was approved by the Institute Research Ethics Committee at Cancer Center (The Fourth Hospital of Harbin Medical University).

### RNA isolation and qRT-PCR

Total RNA of tissue samples or cells were isolated using TRIzol Reagent (Invitrogen) and cDNA was synthesized according to the manufacturer's protocol (MBI Fermentas). MicroRNAs were quantitated by real-time PCR using TaqMan MicroRNA assay (Invitrogen, USA). The 20 μl PCRs reactions included 1 μl of RT product, 1 Universal TaqMan Master Mix and 1xTaqMan probe/primer mix (Invitrogen, USA, Table S2). For miRNAs, U6 snRNA was used as the endogenous control. For mRNA, GAPDH was used as the endogenous control.

### Oligonucleotide transfection

The miR-454 mimics, miR-454 inhibitor, control and the scramble mimics were synthesized by GenePharma (Shanghai, China) and transfected into the cells to a final oligonucleotide concentration of 20 nmol/L. All cell transfections were introduced by DharmaFECT1 Reagent (Dharmacon, TX, USA) following to the manufacturer's instructions.

### Cell proliferation assay

Cell proliferation was conducted using Cell Counting Kit-8 (Dojindo, Kumamoto, Japan). In accordance with the manufacturer's instructions, cells were seeded in 96-well plates in a final volume of 100 μL. Ten μL CCK-8 solutions were added into each well, and the absorbance at 450 nm was measured after incubation for 2 hours at 37°C to measure the number of viable cells.

### Cell invasion assay

The cell invasion assay was measured using BD BioCoat Matrigel invasion chambers (BD Biosciences, Heidelberg, Germany) according the manufacturer's protocol. In brief, 24 h after transfection, cells in 0.5 ml serum-free medium were seeded into the top chamber with a Matrigel coated filter and 0.75 ml DMEM containing 10% FBS was used as a chemoattractant. After incubation, cells that migrated to the lower side were fixed and stained with 1% toluidine blue in 1% borax solution. Stained cells were counted and pictures were captured (Leica, Solms, Germany).

### Luciferase activity assay

Luciferase reporter gene assay was performed using the Dual-Luciferase Reporter Assay System (Promega) following to the manufacturer's instructions. Cells of 90% confluence were seeded in 96-well plates. For AKT2 30-untranslated region (UTR) luciferase reporter assay, wild-type or mutant reporter constructs (termed WT or Mut) were cotransfected into HepG2 cells in 96-well plates with 100 nmol/L miR-454 or 100 nmol/L miR-NC and Renilla plasmid by using lipofectamine 2000 (Invitrogen). Reporter gene assays were performed 24 hours posttransfection using the Dual-Luciferase Assay System (Promega). Firefly luciferase activity was normalized for transfection efficiency using the corresponding Renilla luciferase activity.

### Western blot analysis

Western blot analysis was performed using standard methods. Protein lysates from cells were subjected to 10% SDS-PAGE and target proteins were detected with primary antibodies Ki-67 (Abcam, Cambridge, MA, USA), CHD5, E-cadherin, N-cadherin, Snail and vimentin (Sigma-Aldrich) and GAPDH (Cell Signaling), respectively. After incubation with appropriate horseradish peroxidase (HRP)-conjugated secondary antibodies (Jackson ImmunoResearch), protein bands were visualized using enhanced ECL plus Western blotting detection reagents followed by exposure to Hyperfilms (Amersham, Buckinghamshire, UK).

### Animal studies

Animal studies were done following to institutional guidelines; HepG2 cells were injected subcutaneously into the posterior flanks of 6 week female nude mice. When tumor size reached 50 mm3, miR-454 inhibitor or control were injected into the tumors, respectively. These tumors were injected every 3 days for a total of six times. Tumors were collected and weighted after necropsy.

### Statistical analysis

Data were presented as the mean ±SD from three separate experiments. The differences between groups were analyzed using Student's *t*-test when only two groups were compared or a one-way analysis of variance (ANOVA) when more than two groups were compared. The differences between groups of metastasis *in vivo* were analyzed using the χ2 test. All of the statistical analyses were performed with SPSS 17.0 (SPSS Inc., USA). The difference was considered to be statistically significant at *P* < 0.05.

## SUPPLEMENTARY TABLES


