# Supplementary material for: miR-454 functions as an oncogene by inhibiting CHD5 in hepatocellular carcinoma
Source: Oncotarget. 2015 Jul 30;6(36):39225–34. doi: 10.18632/oncotarget.4407 (PMC4770768; doi:10.18632/oncotarget.4407)
Supplement: Supplementary file 1 [file oncotarget-06-39225-s001.pdf]

SUPPLEMENTARY TABLES

Supplementary Table S1. Summary of clinicopathological parameters of patients with hepatocellular carcinoma

	Clinicopathological parameters	Number of cases
Gender	≥55	27
	<55	23
Age	Male	36
	Female	14
With cirrhosis	Present	33
	Absent	17
TNM stage	I, II	35
	III, IV	15

Supplementary Table S2. Primer sequence

Name	Sequence (5′-3′)
miRNA-709	TGCGGGGAGGCAGAGGCA
	CCAGTGCAGGGTCCGAGGT
U6 snRNA	TGCGGGTGCTCGCTTCGGCAGC
	CCAGTGCAGGGTCCGAGGT
GAPDH	GGTGAAGGTCGGTGTGAACG
	CTCGCTCCTGGAAGATGGTG
CHD5	TGCTTAAAGGAGCCCAAGTC
	TTGGTCAGCGTGTGGTAATC
Ki-67	TCCTTTGGTGGGCACCTAAGACCTG
	TGATGGTTGAGGTCGTTCCTTGATG
